# Concurrent Validity of Power From Three On-Water Rowing Instrumentation Systems and a Concept2 Ergometer

**DOI:** 10.3389/fphys.2021.758015

**Published:** 2021-11-12

**Authors:** Ana C. Holt, William G. Hopkins, Robert J. Aughey, Rodney Siegel, Vincent Rouillard, Kevin Ball

**Affiliations:** ^1^Institute for Health and Sport, Victoria University, Melbourne, VIC, Australia; ^2^Sport Science Department, Victorian Institute of Sport, Melbourne, VIC, Australia; ^3^Australian Institute of Sport, Canberra, ACT, Australia; ^4^College of Engineering and Science, Victoria University, Melbourne, VIC, Australia

**Keywords:** Peach PowerLine, Weba OarPowerMeter, Nielsen-Kellerman EmPower, technical error of measurement, systematic error, between-unit differences, Concept2, random error

## Abstract

**Purpose:** Instrumentation systems are increasingly used in rowing to measure training intensity and performance but have not been validated for measures of power. In this study, the concurrent validity of Peach PowerLine (six units), Nielsen-Kellerman EmPower (five units), Weba OarPowerMeter (three units), Concept2 model D ergometer (one unit), and a custom-built reference instrumentation system (Reference System; one unit) were investigated.

**Methods:** Eight female and seven male rowers [age, 21 ± 2.5 years; rowing experience, 7.1 ± 2.6 years, mean ± standard deviation (SD)] performed a 30-s maximal test and a 7 × 4-min incremental test once per week for 5 weeks. Power per stroke was extracted concurrently from the Reference System (*via* chain force and velocity), the Concept2 itself, Weba (oar shaft-based), and either Peach or EmPower (oarlock-based). Differences from the Reference System in the mean (representing potential error) and the stroke-to-stroke variability (represented by its SD) of power per stroke for each stage and device, and between-unit differences, were estimated using general linear mixed modeling and interpreted using rejection of non-substantial and substantial hypotheses.

**Results:** Potential error in mean power was decisively substantial for all devices (Concept2, –11 to –15%; Peach, −7.9 to −17%; EmPower, −32 to −48%; and Weba, −7.9 to −16%). Between-unit differences (as SD) in mean power lacked statistical precision but were substantial and consistent across stages (Peach, ∼5%; EmPower, ∼7%; and Weba, ∼2%). Most differences from the Reference System in stroke-to-stroke variability of power were possibly or likely trivial or small for Peach (−3.0 to −16%), and likely or decisively substantial for EmPower (9.7–57%), and mostly decisively substantial for Weba (61–139%) and the Concept2 (−28 to 177%).

**Conclusion:** Potential negative error in mean power was evident for all devices and units, particularly EmPower. Stroke-to-stroke variation in power showed a lack of measurement sensitivity (apparent smoothing) that was minor for Peach but larger for the Concept2, whereas EmPower and Weba added random error. Peach is therefore recommended for measurement of mean and stroke power.

## Introduction

Rowing instrumentation systems provide a comprehensive measure of performance given their ability to assess both technical and physical components of rowing performance and enable instantaneous quantitative feedback to the rower (Lintmeijer et al., 2019; Holt et al., 2020). Instantaneous feedback of power output has been shown to improve training intensity adherence by 65% in rowers compared to boat velocity, stroke rate, and coach feedback alone (Lintmeijer et al., 2019). Furthermore, on-water power measurement in rowing has potential widespread value in the quantification of external training load, analysis of race demands, and performance monitoring *via* power-based benchmarks, all of which have been achieved with instrumentation systems in cycling (Schumacher and Mueller, 2002; Nimmerichter et al., 2011; Sanders and Heijboer, 2019). However, before rowing instrumentation systems can be used with some certainty, their validity must first be established. Knowledge of the systematic error and random error associated with measures of power from rowing instrumentation devices will inform the interpretation of a meaningful change or difference in power for the given device.

Different types of rowing instrumentation systems exist and can be located on at the oarlock or on the oar shaft. Instrumentation systems located on the oarlock measure forces occurring at the pin resulting from the transfer of force applied at the handle to the oar blade. Oar shaft-based instrumentation systems are positioned on the oar’s inboard (the section of oar shaft between the handle and the point of the oar’s rotation at the oarlock) and calculate the moment of force applied to the handle from the deflection of the oar throughout the stroke (Kleshnev, 2010).

The validity of power measurement off-water has been investigated using mechanical sensors attached to Concept2 ergometer models A and D, with negative systematic error estimates of 5–8% reported (Lormes et al., 1993; Boyas et al., 2006). However, the validity of power from on-water instrumentation systems is yet to be established. Research investigating the validity of rowing instrumentation systems has focused on oarlock-based Peach PowerLine devices (Peach Innovations, Cambridge, United Kingdom), encompassing static (Laschowski and Nolte, 2016), or dynamic linear force application and static angle assessment (Coker et al., 2009). Eight Peach sculling units had reasonable concurrent validity for measures of force up to 555 N and angle between −80° to 60°, with standard error of the estimate (SEE) values of 7.16 ± 2.56 N for force and 0.9° ± 0.9° SEE for angle (Coker et al., 2009). Very large correlations between applied and measured forces of up to 432 N were also reported for eight sculling (*r* = 0.985) and nine sweep (*r* = 0.986) Peach units, although a negative error of 2% was observed for Peach (Laschowski and Nolte, 2016). However, the testing methods of these studies do not reflect a rowing-specific pattern of force application or oarlock angular rotation. Force at the oarlock throughout a rowing stroke increases to a peak mid-drive with a subsequent decrease from mid-drive to the end of the drive phase, and therefore do not reflect the static force application used in previous validity studies. Similarly, the measurement of catch and finish oarlock angles during a rowing stroke occur during rotation at each end of the stroke when the oar changes direction, which is not reflected in the static measurement of oarlock angle used previously. As such, the applicability of results from previous validity studies to the measurement of power on-water is unknown. Furthermore, the validity of power measures has not been investigated in Peach or other commercially available rowing instrumentation systems such as the Nielsen-Kellerman EmPower (Kleshnev, 2017) and Weba Sport OarPowerMeter in peer-reviewed research. Therefore, the aim of this study was to assess the concurrent validity of power measures from Peach PowerLine, Nielsen-Kellerman EmPower, Weba Sport OarPowerMeter sweep rowing instrumentation systems, and a Concept2 ergometer with an instrumented Swingulator team sweep system through a dynamic, on-water rowing specific range of oar angles and force applications.

## Materials and Methods

### Participants

Eight female (age 21.6 ± 3.1 years; height 175.9 ± 4.1 cm; and body mass 76.7 ± 5.4 kg, mean ± standard deviation [SD]) and seven male (age 20.9 ± 2.0 years; height 189.7 ± 8.4 cm; and body mass 86.2 ± 11.0 kg) trained rowers with 7.1 ± 2.6 years experience at a national level who were actively participating in the sport at the time of the study and had competed in the previous rowing season volunteered for this study. Six of the participants (five females and one male) had previously represented Australia at International Regattas. Participants provided informed consent prior to commencement of the study. The study was approved by the University Human Research Ethics Committee.

### Equipment

**FIGURE 1:**
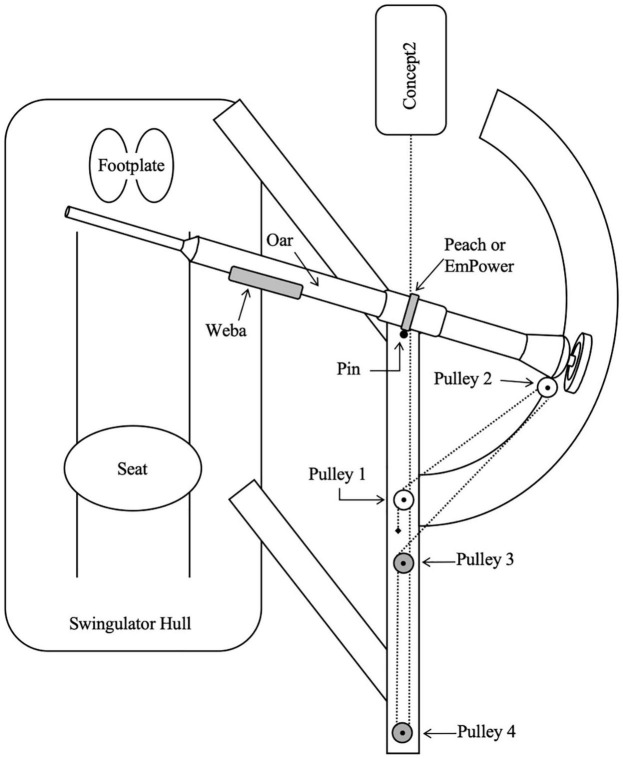
Birds-eye view diagram (not drawn to scale) of Swingulator-system illustrating location of devices. Pin, point of oar rotation. Pulley 2 is attached to the oar, Pulleys 3 and 4 are located on the underside of the Swingulator framing. Dashed line represents the Concept2 chain and Swingulator cord which passes under the framing after Pulley 2. The black diamond near Pulley 1 indicates the anchor point of the Swingulator cord.

All rowing was performed on an instrumented Swingulator team sweep trainer (Rowing Innovations Inc., Williston, VT, United States) with a Concept2 ergometer (Model D with PM5 monitor, Concept2 Inc., Morrisville, VT, United States) attachment. The Swingulator was used as it enables the simulation of on-water sweep rowing in a controlled land-based environment, where power could be recorded from the Reference System, Weba, Concept2, and Peach or EmPower simultaneously. The Swingulator also allowed instrumentation with mechanical sensors (as described in the next paragraph) to provide a comparative measure of power for the assessment of concurrent validity. When using the Swingulator the rower holds the handle of an oar with a shortened outboard which sits in an oarlock, the end of the oar’s outboard (between the oar’s collar and blade-end) connects to a cable which passes through four pulleys before connecting to the chain of the Concept2, which provides resistance during the drive phase of the rowing stroke (Figure 1). Oar inboard (between the oar’s handle and collar) and total oar lengths were set to 114.5 and 177 cm, respectively, with span (distance between the pin and the center of the hull) set to 84.3 cm.

**FIGURE 2:**
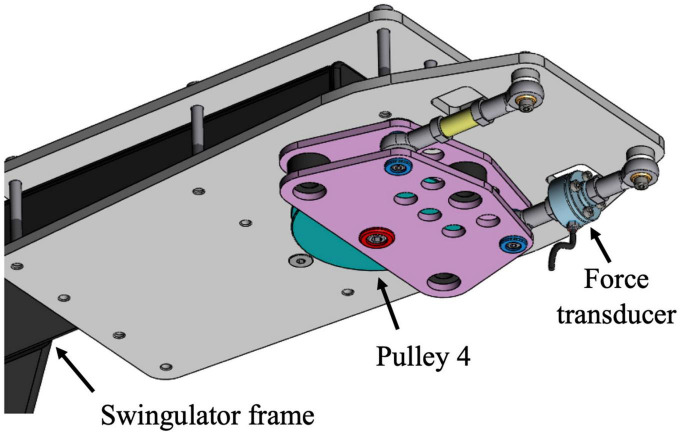
Custom Swingulator attachment with force transducer at Pulley 4.

The Concept2 drag factor was set to 80 units for females and 100 units for males, which were lowered by 30 units from typical settings to account for the greater resistance of the Swingulator-Concept2 system. Mechanical sensors (hereafter referred to as the Reference System) were attached to the Swingulator, similar to that used previously on Concept2 ergometers (Macfarlane et al., 1997; Boyas et al., 2006), and included a quadrature optical encoder (HEDS-5500 Optical Encoder) coupled inline to the Concept2’s chain, allowing finite linear displacement to be measured with regard to a fixed reference mark. A force transducer (DACELL UMMA-K200) was housed in a custom attachment (Küsel Dësign, Melbourne, VIC, Australia) at Pulley 4 on the Swingulator assembly (Figure 2), enabling the measurement of force applied through the Swingulator cord when a participant pulled on the oar handle. Static testing of the force transducer without attachment to the Swingulator against a known mass between 0.2 and 85.8 kg was undertaken to verify the linearity characteristics and the voltage-force-mass relationship, which had an *R*-squared (*R*^2^) value of 1.00. The quadrature optical encoder was assessed in pilot testing using a Vicon analysis system (T-40 series, Vicon Nexus v2.7, Oxford, United Kingdom) with a 14-mm diameter reflective marker attached to the Concept2 chain. An *R*^2^ value of 0.99 was found between the quadrature optical encoder and the Vicon analysis system for the measurement of Concept2 chain displacement.

Calibration of the Reference System’s force and displacement measurement on the Swingulator was performed every 7 days throughout the study. For force calibration, the oar handle was locked perpendicular to the Swingulator’s hull and loaded with a known mass (1000.4 N; 101.98 kg). Calibration of the quadrature optical encoder was achieved by movement of the chain through its full range on the Swingulator (∼2030 mm). Although calibration of the Reference System could not be performed prior to each testing session due to the timeframes involved, the analysis allowed the error introduced between sessions that was associated with the Reference System to be partitioned out from that introduced by the other devices, and is reported in the results section.

The concurrent validity of power from Peach PowerLine (Peach Innovations, Cambridge, United Kingdom), EmPower (Nielsen-Kellerman, Boothwyn, PA, United States), Weba (OarPowerMeter, Weba Sport, Wien, Austria) sweep instrumentation devices and the Concept2 ergometer were tested. Differences in concurrent validity between different units for each device was also assessed through testing of six Peach units, five EmPower units, and three Weba units. Peach and EmPower units were attached to the Swingulator’s pin, replacing the oarlock. Peach and EmPower baseplates were attached to the pin at 90° to the Swingulator’s hull (as per manufacturer’s instructions) using a straight edge and goniometer (EZ Read, Jamar, Performance Health, IL, United States). Weba devices were placed facing the participant on the inboard of the oar shaft, as per manufacturer’s instructions.

Calibration procedures for Peach, EmPower, and Weba devices were performed in accordance with manufacturer instructions immediately prior to each testing session. Calibration of the oarlock angle for Peach and EmPower devices was achieved using a goniometer and straight edge to set the unit’s angle as 0° when the oarlock’s flat edge was 90° to the Swingulator’s hull. An additional angle calibration routine was performed for EmPower units using the calibration tool supplied by the manufacturer, successful calibration for this additional process was determined by the unit itself. Force for Peach and EmPower devices was calibrated *via* zeroing the unit’s force measure with the oar removed. Calibration of Weba devices was achieved *via* the hanging of a known mass (198.3 N; 20.22 kg) ∼10 cm from the handle tip with the oar’s outboard held in a horizontal position on a bench with the Weba unit facing downward.

### Testing Protocol

**FIGURE 3:**
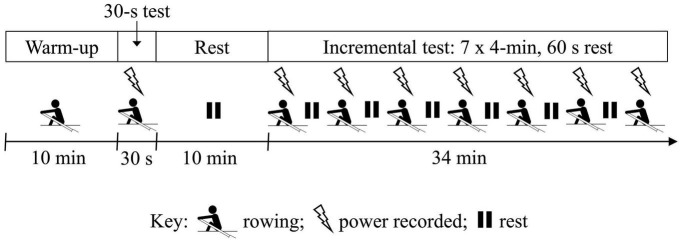
Schematic of testing session protocol showing periods of rowing (rower icon), rest (pause icon), and when power was recorded from the five devices (lightning icon). Participants performed the same protocol in each of the five testing sessions, which were separated by 7 days.

The study was conducted in a temperature-controlled environment (21.1 ± 1.0°C; 48.6 ± 9.9% RH). Participants performed five testing sessions on the Swingulator team sweep trainer separated by 7.0 ± 2.0 days, including one initial familiarization session. A schematic of the testing session procedures is illustrated in Figure 3. Testing sessions included a 10-min warm-up of low-intensity rowing interspersed with three maximal 10-stroke efforts, then a maximal 30-s rowing test at a self-selected stroke rate. Following a subsequent 10 min rest period participants undertook a 7 × 4-min incremental test at self-selected stroke rates, including a final maximal 4-min stage (Tanner and Gore, 2013). A 60-s recovery period was performed between each stage of the incremental test. Participants were instructed to maintain a prescribed power for Stages 1–6 of the incremental test, which were individualized based on the participants most recent 2000-m ergometer test (Tanner and Gore, 2013), and adjusted to account for the perceived resistance of the Swingulator in comparison to rowing on a standard Concept2 ergometer. The familiarization session further guided the prescription of prescribed power for Stages 1–6, which remaining constant across the final four testing sessions. Participants were instructed to row full-length strokes for the 30-s test and throughout the 7 × 4-min test. The 30-s maximal and 7 × 4-min incremental tests were selected for the assessment of concurrent validity as they are performed as part the participants’ regular rowing testing and were therefore familiar to participants, and provided measures of power across intensities ranging from very low to maximal.

The Reference System, Concept2, and Weba were tested concurrently for all testing sessions. The use of Peach or EmPower was alternated per testing session, with each participant performing either two or three testing sessions with each device. The testing order of Weba, Peach, and EmPower units was randomized. Peach units were tested in a total of 37 testing sessions, including 20 sessions on bow side and 17 sessions on stroke side, with individual units assessed in 2–8 sessions each. EmPower units were tested in a total of 38 testing sessions, including 20 sessions on bow side and 18 sessions on stroke side, with individual units assessed in 4–9 sessions each. Weba units were tested in a total of 75 testing sessions, including 40 sessions on bow side and 35 sessions on stroke side, with individual units assessed in 23–27 sessions each.

### Data Analysis

Power per stroke was recorded for Peach and EmPower by their respective head units and exported to a comma-separated values (CSV) file. Power per stroke for Weba was recorded on a Lenovo Tab 4 8 tablet (Lenovo Group Ltd., Beijing), data could not be exported from the tablet so the tablet’s screen was recorded and power per stroke manually entered into Microsoft Excel with the manually entered data checked against the recording for input errors. Power per stroke was recorded from the Concept2 using the app PainSled (version 1.1.0, Charlotte Intellectual Properties, LLC, Charlotte, NC, United States) and exported to CSV files.

Chain displacement and force from the Reference System was recorded at 271.7 Hz and filtered in MATLAB (R2019b, The MathWorks, Inc., Natick, MA, United States) using a low-pass fourth order Butterworth filter with cut-off frequencies of 6 and 13 Hz for displacement and force data, respectively. The choice of cut-off frequencies were informed by residual analysis (Winter, 1990) and supported by visual inspection of raw and smoothed curves.

**FIGURE 4:**
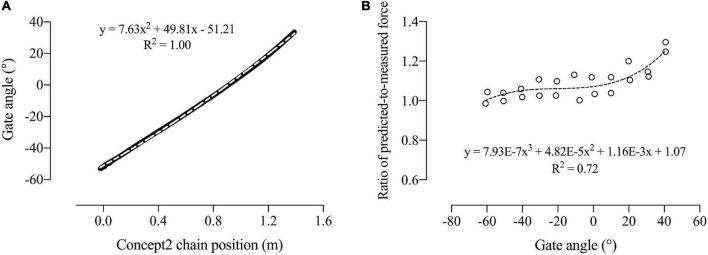
Oarlock angle plotted against Concept2 chain position used to derive *a*, *b*, and *c* in Equation 1 **(A)**; and the ratio of predicted-to-measured force plotted against oarlock angle used to derive the coefficients *d*, *e*, *f*, and *g* in Equation 4 **(B)**. Dashed lines represent the fitted trendlines.

The corresponding oarlock angle for a given chain position was calculated using chain position and oarlock angle data collected by the Vicon motion analysis system in previous testing of the Swingulator-Concept2 system. Oarlock angle was plotted over chain position during the drive phase of the stroke (between the maximal negative oar angle and the subsequent maximal positive oar angle per stroke), and fitted with a second order polynomial trendline that had an *R*^2^ value of 1.00 (Figure 4A) to derive *a*, *b*, and *c*, in Equation 1:


(1) $$P_{C}=P_{M}-\left(\frac{b+\left(\sqrt{b^{2}-4\cdot a\cdot \left(c-\theta \right)}\right)}{2\cdot a}+P_{M}\right)$$

where *P*_*C*_ is the corrected chain position, *P*_*M*_ is the measured chain position, *b* is 49.81, *a* is 7.63, *c* is −51.21, and *θ* is the initial oarlock angle at rest.

The calculated oarlock angle (*θ_*C*_*) was then derived by:


(2) θC=a⋅PC2+b⋅PC-c

**FIGURE 5:**
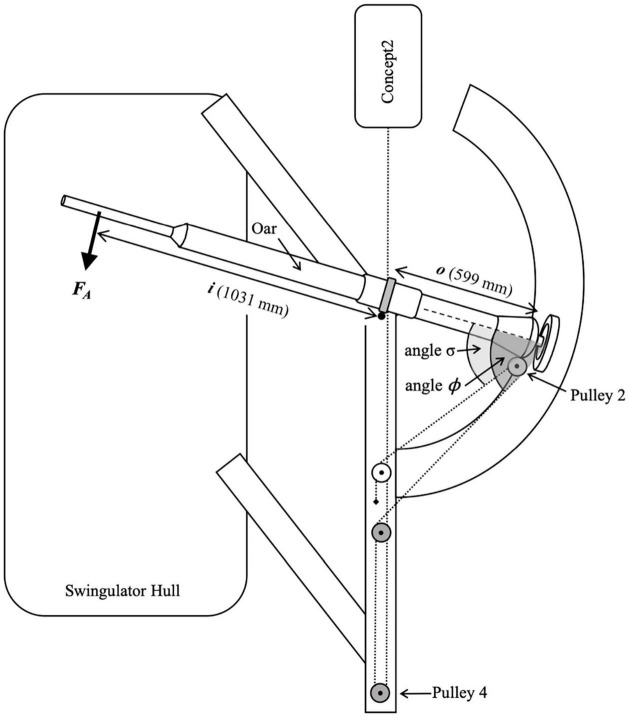
Free body diagram of Swingulator-system illustrating the location of the applied force (*F*_*A*_), distance *i*, distance *o*, angle *σ*, and angle ϕ in Equation 3.

Due to the geometry of the Swingulator system, force measured at Pulley 4 was corrected relative to the calculated oar angle. A static force of 198.4 N was applied to the oar handle at 11 oarlock angles ranging between 40.75° and −60.75° on each of bow and stroke sides. First force was predicted (*F*_*P*_) using Equation 3:


(3) $$F_{P}=\frac{0.5\cdot \left(i/o\right)\cdot F_{A}}{c\,o\,s\cdot \left(90-\left(\sigma +\phi \right)/2\right)}$$

where *i* was the distance between the pin and the point of force application at the oar handle (1031 mm), *o* was the distance between the pin and the point on the oar that aligned with the center of Pulley 2 (599 mm), *F*_*A*_ was the force applied to the oar’s handle (198.4 N), *σ* is the angle between the oar shaft and the Swingulator cord on the inner (Figure 5) side of Pulley 2, and ϕ is the angle between the oar shaft and the Swingulator cord on the outer side of Pulley 2.

The ratio of predicted-to-measured force was then calculated, plotted against oarlock angle, and fitted with a third order polynomial trendline that had an *R*^2^ value of 0.72 (Figure 4B) to derive *d*, *e*, *f*, and *g* in Equation 4 for corrected force (*F*_*C*_):


(4) FC=FA×(d⋅θC3+e⋅θC2+f⋅θC+g)

where *θ_*C*_* is the calculated oarlock angle from Equation 2, *d* is 7.93E−7, *e* is 4.82E−5, *f* is 1.16E−3, and *g* is 1.07.

Instantaneous work (*W*_*i*_) was then calculated from *P*_*C*_ and *F*_*C*_ for each sample:


(5) $$W_{i}=\left(\frac{F_{C\,1}+F_{C\,2}}{2}\right)\times \left(P_{C\,2}-P_{C\,1}\right)$$

where *F*_*C*__1_ and *P*_*C*__1_ indicate the previous data point and *F*_*C*__2_ and *P*_*C*__2_ indicate the current data point.

Power per stroke was calculated using Visual 3D (version 6.3, C-Motion, Inc., Germantown, MD, United States) from stroke work over stroke time. Stroke time was calculated as the number of samples between consecutive finish positions (the maximum *P*_*C*_ per stroke) multiplied by 0.00368 s (corresponding to the sample rate of 271.7 Hz). Stroke work was calculated as the integral of *W*_*i*_ over the drive phase (from the catch to finish position of the current stroke). Power per stroke was then calculated as stroke work divided by stroke time and exported from Visual 3D into Microsoft Excel where it was aligned by stroke number with power from Peach or EmPower, Weba, and the Concept2 for each stage.

The first and last two strokes per stage were excluded from analyses from each device to eliminate some inconsistencies between the devices at the onset and termination of rowing. Outliers defined as strokes where power was greater than seven SDs from the stage mean power for that device were also excluded; 24 such outliers were identified, but only for Weba units. The magnitude of seven SD was chosen based on time series graphs and represented visually obvious outliers that might prompt the practitioner to disregard the data or repeat the test. This value was chosen as a reasonable compromise between removing data that clearly should be excluded, but not removing data that would not have been visually obvious to practitioners when using devices in the field. Occasionally, errors relating to the recording of devices (both human and device errors) resulted in missing strokes or whole stages for certain device units. Stages where a device had more than five missing strokes were excluded from the analyses for that device. A total of 14 stages were missing or excluded for the Reference System, which were also excluded for the other devices. The number of additional stages missing data or excluded due to missing strokes were 3 for Peach, 1 for EmPower, 6 for Weba, and 32 for the Concept2 (of which the stages excluded due to missing data were split between the 30-s test and Stage 7 and were related to not all strokes being recorded successfully by the PainSled app). After the exclusion of stages with missing data, the mean percentage of strokes missing from analyses (including those excluded as outliers) in each stage were: ≤0.1% for the Reference System and EmPower; ≤0.2% for Peach; ≤0.4% for Weba Stages 1–7, but 2.2% for the 30-s test; and ≤0.5% for the Concept2 Stages 1–7, but 6.5% for the 30-s test. Following the exclusion of these strokes and stages, additional outliers were identified as stages with a standardized residual greater than 4 after running the model (Hopkins et al., 2009). Two Weba stages were identified as outliers from the analysis of mean power, and eight stages (one for the Concept2, five for EmPower, and two for Weba) were identified as outliers from the analysis of the SD of power. All stages identified as outliers were included in the analyses as such data would not be identifiable and therefore not removed when devices are used in the field. The data analyzed in this study is available in an online repository (see Data Availability Statement).

### Statistical Analysis

**FIGURE 6:**
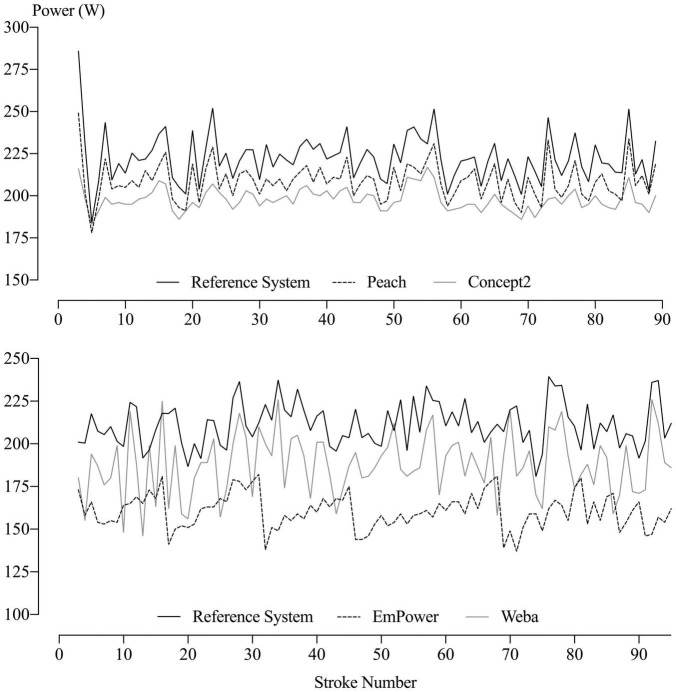
Power per stroke for each device during Stage 5 of two consecutive testing sessions by the same participant. Data for the Weba and Concept2 were recorded in every session but for reasons of clarity are not shown for the Weba (above) or the Concept2 (below).

Although the data consisted of individual values of mean power for each stroke from each of the five devices, the values for EmPower, Weba, and the Concept2 could not be aligned reliably with those of the Reference System, as can be seen in an example of the data from one stage in Figure 6. A repeated-measures analysis of the individual stroke values was therefore not possible. Instead, the mean power for the 30-s stage and for each stage of the incremental test was analyzed with a mixed model, using a separate analysis for each stage. The same model was applied to the SD of power for each stage, representing the stroke-to-stroke variability in power within a stage (note: this is a lengthy section with detailed statistical methods and might be skipped by the applied reader).

The general linear mixed-model procedure (Proc Mixed) was used to perform the analysis in the Studio On-demand for Academics edition of the Statistical Analysis System (version 9.4, SAS Institute, Cary NC, United States). The dependent variable was the log of the mean and the log of the log of the factor SD. The fixed effects were device identity and device identity interacted with Reference System power to estimate, respectively, systematic error (representing the difference in mean power from the Reference System for a given device) and proportional error (representing the change in mean error for a given change in the Reference System’s mean power or the Reference System’s stroke-to-stroke variability in power) for each device. With these fixed effects, a separate residual for each device (estimated as a variance and expressed as an SD) was specified to represent technical error of measurement (TEM) (random session-to-session changes in error) of the device. Random effects of increasing complexity were added to the model to account for and reduce what turned out to be substantial residual variance. The final random effects (estimated as variances and expressed as SD) were: device identity interacted with unit identity (representing differences in error between units for Weba, Peach, or EmPower devices; a separate variance was estimated for each device); session identity (representing differences between the testing sessions that were experienced equally by the three devices in the given session and therefore potentially changes in the Reference System between sessions); and participant identity (representing differences between participants; a separate variance was estimated for each device, to allow for each device responding uniquely to each participant’s rowing style). A random effect representing differences in proportional error between each unit of each device was also investigated; the effect was unclear for all devices but consistent with trivial for Peach and Weba, so this effect was not included in the final model. For the analysis of the SDs (stroke-to-stroke variations), random effects representing differences between participants and potential session-to-session differences arising from the Reference System are not presented, but are available on request, as are the residuals representing the TEM for the SDs of each device.

Plots of residuals vs. predicteds were examined for outliers and evidence of non-uniformity. To ensure correct interpretation of the random effects and residuals, analyses of mean power were also performed by including data for an additional device simulating the Peach: the data for this device were those of the Reference System, but with added random error of 5% for each session, and with five units simulated by adding 3, 6, 9, 12, and 15% to the Reference System values. Finally, to investigate the extent to which changes in error in each device between sessions (evident as the residuals) arose from random changes in the device, mean correlations of the residuals of each device with the other devices were computed for each stage (expected values of 0.00, if the session-to-session error arose entirely separately in each device), and mean correlations of the residuals of each stage with the other stages were computed for each device (expected values approaching 1.00, if the session-to-session error in each device was consistent across the stages in a given session).

A smallest substantial change in power of 1.0% was assumed from the 1.0% race-to-race variation in 2000-m race times of elite rowers (corresponding to a 0.3% smallest substantial change in rowing velocity) and the assumption that power is proportional to velocity cubed (Smith and Hopkins, 2011). Corresponding magnitude thresholds were based on the factors for competitive performance (Hopkins et al., 2009) and are used to provide a practical description of the magnitude of error relative to the magnitude for a meaningful change in performance (i.e., the smallest substantial change in power); for positive changes in power these were <1.0% trivial, ≥1.0% small, ≥3.0% moderate, ≥5.5% large, ≥8.6% very large, and ≥14% extremely large; for negative changes the thresholds were >−1.0% trivial, ≤−1.0% small, ≤−2.9% moderate, ≤−5.2% large, ≤−8.0% very large, and ≤−12% extremely large. To evaluate the magnitudes of SDs representing between-unit differences in mean power and the residuals, the magnitude thresholds were one-half of those in the above scales (Smith and Hopkins, 2011): <0.5% trivial, ≥0.5% small, ≥1.5% moderate, ≥2.7% large, ≥4.2% very large, and ≥6.7% extremely large. Magnitudes of proportional error were assessed for a 10% difference in Reference System mean power; the usual two between-subject SDs (Hopkins et al., 2009) was not appropriate, given the wide range in power between participants arising from the inclusion of males and females.

Magnitude thresholds for comparing the mean SDs of power (representing the mean stroke-to-stroke variability in power within a stage) were the usual factor thresholds for hazards and counts (Hopkins et al., 2009); for factor increases (which would occur when a device adds noise to the participant’s stroke-to-stroke variability, as demonstrated by Weba in comparison to the Reference System in Figure 6) the thresholds were <1.11 (11%) trivial, ≥1.11 small, ≥1.43 (43%) moderate, ≥2.0 (100%) large, ≥3.3 (230%) very large, and ≥10 (900%) extremely large; for factor decreases (which would occur when a device lacks measurement sensitivity for stroke-to-stroke variations in power, as demonstrated by the Concept2 in comparison to the Reference System in Figure 6) the thresholds were >0.90 (−10%) trivial, ≤0.90 small, ≤0.70 (−30%) moderate, ≤0.50 (−50%) large, ≤0.30 (−70%) very large, and ≤0.10 (−90%) extremely large. To evaluate the magnitudes of SDs representing between-unit differences in the mean SD in power, the magnitude thresholds are one-half of those for factor increases: <1.05 (5.4%) trivial, ≥1.05 small, ≥1.2 (20%) moderate, ≥1.41 (41%) large, ≥1.83 (83%) very large, and ≥3.16 (220%) extremely large. Magnitudes of proportional error were assessed for a two between-subject SD in the Reference System mean SD of power (Hopkins et al., 2009), because gender differences were not expected to affect between-subject differences in the SD expressed in percent units.

The thresholds for comparing the SDs were justified by using simulation (with spreadsheets) to investigate the extent to which noise and a loss in measurement sensitivity (apparent smoothing) for power per stroke modify effects involving power per stroke as either a predictor or a dependent variable. The effect of power per stroke as a predictor with added noise is attenuated by a factor equal to the square of the ratio of the SD of true power per stroke (represented by the Reference System) divided by the SD of the predictor (represented by the device), when the effect of the predictor is expressed per unit of the predictor (as stated by Hopkins et al., 2009); however, when expressed per 2 SD of the predictor, the effect is attenuated by the ratio of the SDs without squaring. Effects when power per stroke is a predictor with a lack of measurement sensitivity are *increased* by a factor equal to the ratio of the SD of true power per stroke divided by the SD of the predictor, when the effect of the predictor is expressed per unit of the predictor; however, when expressed per 2 SD of the predictor there is negligible attenuation of the effect (<5%) when the ratio of measured/true SD is >0.7. For effects when power per stroke is a dependent variable with added noise, there is no modification of the effect magnitude (as stated by Hopkins et al., 2009). Effects when power per stroke is a dependent variable with a lack of measurement sensitivity are attenuated by a factor equal to the ratio of the SD of the dependent variable divided by the SD of true power per stroke, when the ratio is >0.7; for a reduction in measurement sensitivity (e.g., ratio of SDs = 0.6), the attenuation is a little greater than the ratio (0.65). In summary, noise or a lack of measurement sensitivity of power per stroke does not modify effects in two scenarios, but it modifies effects by factors given by the ratio of the SDs in three scenarios (effect attenuation in two, effect amplification in one) and by the square of the ratio in one scenario. We therefore opted to assess the effect of noise and a lack of measurement sensitivity by assessing the ratio of the SDs, and we used the thresholds for ratios of hazards and counts, since it seems reasonable to consider that modifications of an effect magnitude by a factor of 0.9 (or its inverse, 1.11) through to 0.1 (or its inverse 10) represent thresholds for small through to extremely large. Researchers should be aware that square roots of these thresholds will apply to a noisy predictor per unit of the predictor, but that there is no effect on the magnitude per 2 SD of a predictor with a modest lack of measurement sensitivity, and no effect on magnitude with a noisy dependent.

Sampling uncertainty in the estimates of effects is presented as 90% compatibility limits in the tables. For those who prefer a frequentist interpretation of sampling uncertainty, decisions about magnitudes accounting for the uncertainty were based on one-sided interval hypothesis tests, where an hypothesis of a given magnitude (substantial, non-substantial) was rejected if the 90% compatibility interval fell outside that magnitude (Aisbett et al., 2020; Hopkins, 2020). *p*-Values for the tests were the areas of the sampling distribution of the effect (t for means, *z* for random-effect variances, Chi-squared for residual variances) falling in the hypothesized magnitude, with the distribution centered on the observed effect. Hypotheses of inferiority (substantial negative) and superiority (substantial positive) were rejected if their respective *p*-values (*p*_–_ and *p*_+_) were <0.05; rejection of both hypotheses represents a decisively trivial effect in equivalence testing. For residual variances, only the tests of superiority and non-superiority were relevant. When only one hypothesis was rejected, the *p*-value for the other hypothesis, when >0.25, was interpreted as the posterior probability of a substantial true magnitude of the effect in a reference-Bayesian analysis with a minimally informative prior (Hopkins, 2019) using the following scale: >0.25, possibly; >0.75, likely; >0.95, very likely; >0.995, most likely (Hopkins et al., 2009); the probability of a trivial true magnitude (1 – *p*_–_ – *p*_+_) was also interpreted, when >0.25, with the same scale. Probabilities were not interpreted for effects that were unclear (those with inadequate precision at the 90% level, defined by failure to reject both hypotheses, *p*_–_ > 0.05 and *p*_+_ > 0.05). Effects with adequate precision at the 99% level (*p*_–_ < 0.005 or *p*_+_ < 0.005) are in bold in the tables; these represent effects that have a conservative low risk of error or noise. The hypothesis of non-inferiority (non-substantial-negative) or non-superiority (non-substantial-positive) was rejected if its *p*-value (p_*N*__–_ = 1 – *p*_–_ or p_*N*__+_ = 1 – *p*_+_) was <0.05, representing a decisively substantial effect in minimal-effects testing: very likely or most likely substantial.

## Results

The power per stroke throughout a stage for the five devices is exemplified in Figure 6. The mean and SD (representing the stroke-to-stroke variation in power) of the individual values of power per stroke in Figure 6 for each device (along with those of all the other stages and testing sessions) provided the data for the subsequent analyses.

**FIGURE 7:**
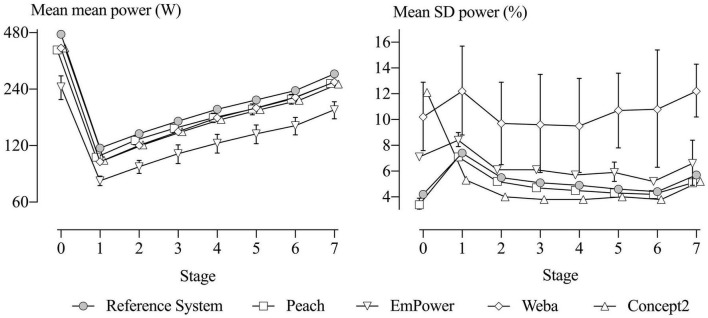
Means of the mean (left) and SD (right) of power for each device in each stage for all the testing sessions. SD bars represent between-unit SD for the means and SD. The Reference System and the Concept2 have no SD bars, as only one unit was tested. SD bars on the right are omitted from some stages for Peach and EmPower, reflecting negative variance. Stage 0 represents the 30-s maximal test.

The mean power of each of the five devices across all testing sessions and participants for each stage is shown in Figure 7 (left), with SD bars representing between-unit differences in the mean power. The mean stroke rates performed for each stage were: 30-s test, 43.8 ± 7.0 (stroke min^–1^, mean ± SD); Stage 1, 18.0 ± 1.0; Stage 2, 19.4 ± 1.0; Stage 3, 21.2 ± 1.2; Stage 4, 23.0 ± 1.4; Stage 5, 24.8 ± 3.0; Stage 6, 27.3 ± 1.7; and Stage 7, 32.7 ± 1.9.

### Systematic Error

The difference in mean power from the Reference System (representing systematic error) across the eight stages were all decisively substantial and negative (rejection of the non-inferiority hypotheses, p_*N*__–_ < 0.05), and are presented in Table 1. Magnitudes were very large in most stages for Peach, extremely large for EmPower, and mostly very large to extremely large for Weba and the Concept2. Within each device, there is a consistent percent error across Stages 1–7, as shown by the near-equal spacing from Reference System values with a log scale on the *y*-axis in Figure 7. All devices showed relatively greater systematic error in the 30-s test.

**TABLE 1:** Mean systematic error across the stages for Peach, EmPower, Weba, and the Concept2. Data are mean (%), ±90% compatibility limits, with observed magnitude and *p*-values for inferiority and superiority tests (*p*_–_/*p*_+_).

	**Peach**	**EmPower**	**Weba**	**Concept2**
30-s test	–16.9, ±2.7; e.large**** >0.999/<0.001	–47.7, ±8.1; e.large**** 0.999/0.001	–15.4, ±3.9; e.large**** 0.996/0.003	–15.2, ±0.8; e.large**** >0.999/<0.001
Stage 1	–10.5, ±3.4; v.large**** 0.999/0.001	−32.3, ±9.1; e.large*** 0.98/0.02	−15.5, ±6.0; e.large*** 0.99/0.01	–13.9, ±1.6; e.large**** >0.999/<0.001
Stage 2	–7.9, ±4.3; Large*** 0.99/0.005	–32.9, ±7.1; e.large*** 0.995/0.005	–13.4, ±3.9; e.large**** 0.996/0.003	–13.0, ±1.7; e.large**** >0.999/<0.001
Stage 3	–8.8, ±4.0; v.large*** 0.995/0.002	−32.2, ±9.5; e.large*** 0.99/0.01	−12.4, ±4.1; v.large*** 0.99/0.01	–12.3, ±0.6; v.large**** >0.999/<0.001
Stage 4	–10.0, ±4.6; v.large*** 0.99/0.003	−33.2, ±9.5; e.large*** 0.98/0.02	–10.5, ±2.4; v.large**** >0.999/<0.001	–11.9, ±0.7; v.large**** >0.999/<0.001
Stage 5	–11.3, ±5.9; v.large*** 0.99/0.005	−33.6, ±14.3; e.large*** 0.98/0.02	–9.5, ±3.1; v.large**** 0.998/0.001	–11.2, ±0.7 v.large**** >0.999/<0.001
Stage 6	–10.1, ±5.1; v.large*** 0.99/0.003	−34.5, ±9.8; e.large*** 0.99/0.01	–7.9, ±3.0; Large**** 0.996/0.002	–11.3, ±0.6; v.large**** >0.999/<0.001
Stage 7	–11.2, ±4.2; v.large**** 0.998/0.001	−35.4, ±8.5; e.large*** 0.99/0.01	–9.6, ±3.1; v.large*** 0.99/0.004	–11.6, ±0.6; v.large**** >0.999/<0.001

*Scale of magnitudes: >−1%, trivial; ≤−1%, small; ≤−2.9%, moderate (mod); ≤−5.2%, large; ≤−8.0%, very large (v.large); and ≤−12.4%, extremely large (e.large).*

Reference-Bayesian likelihoods of substantial change: *possibly; **likely; ***very likely; and ****most likely.

The symbols *** and **** indicate rejection of the non-superiority or non-inferiority hypothesis (p_*N*__–_ or p_*N*__+_ <0.05 and <0.005, respectively).

*Effects in bold have adequate precision at the 99% level (*p* < 0.005).*

Differences from the Concept2 in mean power across the eight stages are presented in Table 2. Magnitudes were trivial to moderate and mostly positive for Peach, but most were either unclear (the superiority and inferiority hypotheses were not rejected, *p*_+_ and *p*_–_ > 0.05) or were only likely substantial (rejection of only the inferiority hypotheses, *p*_–_ < 0.05). In comparison to the Concept2, mean differences in power for EmPower were negative, extremely large, and decisively substantial. Mean differences in power from the Concept2 for Weba ranged from positive to negative and were trivial to moderate, but most were unclear.

**TABLE 2:** Differences from the Concept2 in mean systematic error across the stages for Peach, EmPower, and Weba. Data are mean (%), ±90% compatibility limits, with observed magnitude and *p*-values for inferiority and superiority tests (*p*_–_/*p*_+_).

	**Peach**	**EmPower**	**Weba**
30-s test	−2.0, ±3.2; Small 0.72/0.06	–38.3, ±9.6; e.large**** 0.998/0.001	−0.2, ±4.3; Trivial 0.35/0.27
Stage 1	3.9, ±4.2; Moderate** 0.03/0.89	−21.4, ±8.0; e.large*** 0.98/0.01	−1.9, ±6.7; Small 0.61/0.20
Stage 2	5.9, ±5.0; Large** 0.02/0.94	−22.9, ±7.9; e.large*** 0.99/0.01	−0.5, ±4.3; Trivial 0.41/0.24
Stage 3	4.0, ±4.6; Moderate** 0.04/0.88	−22.8, ±10.8; e.large*** 0.98/0.02	−0.1, ±4.6; Trivial 0.32/0.28
Stage 4	2.1, ±5.2; Small 0.14/0.66	−24.2, ±12.5; e.large*** 0.97/0.03	1.6, ±2.8; Small 0.06/0.65
Stage 5	0.0, ±6.6; Trivial 0.39/0.38	−25.2, ±15.9; e.large*** 0.96/0.03	1.9, ±3.5; Small 0.08/0.69
Stage 6	1.4, ±5.8; Small 0.23/0.55	−26.2, ±10.9; e.large*** 0.98/0.02	3.8, ±3.4; Moderate** 0.02/0.93
Stage 7	0.4, ±4.8; Trivial 0.29/0.42	−26.9, ±9.6; e.large*** 0.99/0.01	2.2, ±3.3; Small 0.05/0.79

*Scale of magnitudes: <1 and >−1%, trivial; ≥1 or ≤−1%, small; ≥3.0 or ≤−2.9%, moderate (mod); ≥5.5 or ≤−5.2%, large; ≥8.5 or ≤−8.0%, very large (v.large); and ≥14.2 or ≤−12.4%, extremely large (e.large).*

Reference-Bayesian likelihoods of substantial change: *possibly; **likely; ***very likely; and ****most likely.

The symbols *** and **** indicate rejection of the non-superiority or non-inferiority hypothesis (p_*N*__–_ or p_*N*__+_ <0.05 and <0.005, respectively).

*Likelihoods are not shown for effects that were unclear at the 90% level (failure to reject any hypotheses: *p* > 0.05).*

*Effects in bold have adequate precision at the 99% level (*p* < 0.005).*

#### Proportional Error in Systematic Error

Proportional error for a change in power, representing the percentage change in mean error for a 10% change in the Reference System mean power, was estimated for each stage for Peach, EmPower, Weba, and Concept2. Magnitudes of proportional error for a change in power were trivial in most stages for Peach (−2.8% for the 30-s test, and −0.7 to 0.2% for the other stages) and were either unclear or only possibly or likely trivial for most stages. For EmPower, proportional error was trivial to moderate (−4.4% for the 30-s test, and −2.2 to −0.6% for the other stages) and were either unclear or only possibly or likely substantial for most stages. Proportional error was trivial for Weba (−0.6% for the 30-s test, and −0.6 to −0.3% for the other stages) and most effects were possibly or likely trivial. For the Concept2 proportional error was trivial in most stages (0.8% for the 30-s test, and −1.6 to 0.7% for the other stages) with adequate precision that was likely trivial for most stages.

#### Between-Unit Differences in Systematic Error

Between-unit differences in mean power (the SD bars in Figure 7 left) summarize the relative error of the units for a given device, reflecting the degree of differences in systematic error between the units. Only one Concept2 unit was assessed, so no between-unit differences were established for this device. With the exception of one stage for Weba, all the SDs were positive, but unclear. Despite being unclear, the random-effect solutions for the device units (representing the systematic error of each unit) showed evidence of consistent relative error across the stages for most units (data not shown). The observed magnitudes for between-unit differences in mean power were very large in most stages for Peach (an SD of 1.8% for the 30-s test, and 3.6–7.6% for the other stages), extremely large in most stages for EmPower (17% for the 30-s test, and 3.5–9.3% for the other stages), and trivial to large for Weba (1.3% for the 30-s test, and −0.3 to 3.9% for the other stages).

#### Between-Session Error and Between-Participant Error

The SD representing error potentially introduced by the Reference System between sessions were unclear in all but one stage. Observed magnitudes were positive in most stages and small to moderate (−1.6% for the 30-s test, and 1.1–1.8% for the other stages).

Differences in error between participants, estimated as a SD for each device, were unclear for most stages. The observed differences were positive and moderate to very large for Peach (5.0% for the 30-s test, and 2.1–4.5% for the other stages), negative and extremely large for EmPower (−10% for the 30-s test, and −7.0 to −7.8% for the other stages), positive in most stages and small to moderate for Weba (1.6% for the 30-s test, and −0.5 to 4.2% for the other stages), and positive and small in most stages for the Concept2 (0.9% for the 30-s test, and 0.8–3.7% for the other stages).

The residual SD representing the TEM in mean power between sessions (random session-to-session changes in error) was decisively substantial for all stages in Peach, EmPower, Weba, and for most stages for the Concept2, as shown in Table 3. Observed magnitudes were large to very large for Peach, extremely large for EmPower and Weba, and small to large for the Concept2.

**TABLE 3:** Technical (residual) error of measurement representing session-to-session error for mean power recorded by Peach, EmPower, Weba, and the Concept2. Data are SD (%), ±90% compatibility limits, with observed magnitude and *p*-values for the superiority test (*p*_+_).

	**Peach**	**EmPower**	**Weba**	**Concept2**
30-s test	5.1, ±1.7 v.large**** >0.999	26.0, ±9.3 e.large**** >0.999	12.4, ±2.4 e.large**** >0.999	3.5, ±1.2 Large**** >0.999
Stage 1	6.6, ±2.0 v.large**** >0.999	18.6, ±5.7 e.large**** >0.999	11.5, ±2.1 e.large**** >0.999	0.9, ±0.0 Small** 0.90
Stage 2	4.1, ±1.5 Large**** >0.999	18.0, ±5.7 e.large**** >0.999	9.6, ±1.7 e.large**** >0.999	1.3, ±1.5 Small**** >0.999
Stage 3	4.3, ±1.5 v.large**** >0.999	18.6, ±6.7 e.large**** >0.999	9.6, ±1.8 e.large**** >0.999	0.5^a^ Trivial –
Stage 4	4.7, ±1.5 v.large**** >0.999	17.9, ±6.4 e.large**** >0.999	7.6, ±1.4 e.large**** >0.999	1.1, ±3.3 Small*** 0.99
Stage 5	4.5, ±1.7 v.large**** >0.999	18.9, ±7.2 e.large**** >0.999	7.7, ±1.4 e.large**** >0.999	1.4, ±1.3 Small**** >0.999
Stage 6	4.0, ±1.4 Large**** >0.999	17.7, ±6.2 e.large**** >0.999	7.8, ±1.4 e.large**** >0.999	1.6, ±1.1 Moderate**** >0.999
Stage 7	4.8, ±1.7 v.large**** >0.999	17.3, ±6.0 e.large**** >0.999	7.2, ±1.4 e.large**** >0.999	1.4, ±5.8 Small**** 0.997

*Scale of magnitudes: <0.5%, trivial; ≥0.5%, small; ≥1.5%, moderate (mod); ≥2.7%, large; ≥4.2%, very large (v.large); and ≥6.9%, extremely large (e.large).*

*^a^The mixed model failed to produce compatibility limits for this residual.*

Reference-Bayesian likelihoods of substantial change: *possibly; **likely; ***very likely; and ****most likely.

The symbols *** and **** indicate rejection of the non-superiority or non-inferiority hypothesis (p_*N*__–_ or p_*N*__+_ <0.05 and <0.005, respectively).

*Reference-Bayesian likelihoods of trivial change: ^0^possibly; ^00^likely; ^000^very likely; and ^0000^most likely.*

Not shown in the tables are the fixed and random effects for the simulated device, which were used to ensure correct interpretation of the mixed model. The effects for the simulated device, including the residuals, were all consistent with the simulated values. The mean correlations of the technical error residuals of each device with the other devices for each stage ranged from −0.22 to 0.16; this range is consistent with sampling variation when the expected value is 0.00, if the session-to-session error arose entirely separately in each device. The mean correlations of the residuals of each stage with the other stages for each device ranged from 0.92 to 0.98 for the simulated device (where the expected value is 1.00, if the session-to-session error in each device was consistent across the stages in a given session); the ranges for the other devices were: Peach, 0.29–0.77; Empower, 0.73–0.92; Weba, 0.30–0.73; and Concept2, 0.64–0.89. For each device, the lowest mean correlation occurred for the 30-s stage; without this stage the mean correlations were all ∼0.8–0.9, which reflects consistent error across the stages.

### Mean Standard Deviation of Power, Representing Measurement Sensitivity to Stroke-to-Stroke Variations in Power (Random Error)

The mean SD for the Reference System (right in Figure 7) shows that the participants’ true stroke-to-stroke variability in power output was lowest in the 30-s test, highest in Stage 1, declined through Stages 2–6, then increased again for the maximal effort in Stage 7. Differences of each device from the Reference System in the mean SD of power are evident in Figure 7 and are presented in Table 4. Positive differences are consistent with additional noise in stroke-to-stroke variation in power (EmPower and Weba, as demonstrated in comparison to the Reference System in Figure 6), and negative differences are consistent with a lack of measurement sensitivity (Peach and the Concept2, as demonstrated in comparison to the Reference System in Figure 6). Magnitudes were trivial to small and negative for Peach and were possibly, likely, or decisively trivial or substantial. Differences from the Reference System in the SD for EmPower were positive and trivial to moderate and possibly or likely substantial in most stages. For Weba, differences in the SDs from the Reference System were positive, moderate to large and decisively substantial in most stages. The Concept2 showed a positive and large mean SD difference from the Reference System for the 30-s test, but negative and mostly small differences for the other stages that were possibly or decisively substantial.

**TABLE 4:** Differences in the mean SD of power from the Reference System across the stages for Peach, EmPower, Weba, and the Concept2. Data are mean (%), ±90% compatibility limits, with observed magnitude and *p*-values for inferiority and superiority tests (*p*_–_/*p*_+_).

	**Peach**	**EmPower**	**Weba**	**Concept2**
30-s test	–16, ±11; Small** 0.85/0.005	57, ±27; Moderate**** <0.001/0.998	139, ±110; Large*** 0.01/0.98	177, ±46; Large**** <0.001/>0.999
Stage 1	**–3.0, ±4.0; Trivial^0000^** 0.004/<0.001	14, ±16; Small*^0^ 0.02/0.64	61, ±74; Moderate** 0.03/0.93	–27.8, ±3.6; Small**** >0.999/<0.001
Stage 2	**–4.4, ±3.6; Trivial^000^** 0.009/<0.001	9.7, ±5.9; Trivial^0^* <0.001/0.35	73, ±84; Moderate** 0.03/0.94	–27.9, ±4.6; Small**** >0.999/<0.001
Stage 3	**–6.5, ±4.0; Trivial^00^** 0.07/<0.001	20, ±13; Small** <0.001/0.88	88, ±82; Moderate*** 0.02/0.97	–24.3, ±8.2; Small*** 0.99/<0.001
Stage 4	**–6.4, ±5.1; Trivial^00^** 0.11/<0.001	15.5, ±8.4; Small** <0.001/0.81	91, ±78; Moderate*** 0.02/0.97	–20.8, ±9.6; Small*^0^ 0.96/<0.001
Stage 5	**–5.2, ±5.4; Trivial^00^** 0.07/<0.001	29, ±26; Small** 0.09/0.90	126, ±95; Large*** 0.01/0.99	–13.1, ±11.4; Small*^0^ 0.68/0.003
Stage 6	**–3.9, ±5.3; Trivial^000^** 0.03/<0.001	17, ±14; Small** 0.001/0.77	136, ±148; Large*** 0.02/0.97	–13.8, ±10.9; Small*^0^ 0.90/0.01
Stage 7	–9.7, ±6.9; Trivial^0^* 0.47/<0.001	15, ±29; Small 0.05/0.60	109, ±65; Large*** 0.01/0.99	–7.2, ±8.1; Trivial^0^* 0.27/0.002

*Scale of magnitudes: <11 and >−10%, trivial; ≥11 or ≤−10%, small; ≥43 or ≤−30%, moderate (mod); ≥100 or ≤−50%, large; ≥230 or ≤−70%, very large (v.large); and ≥900 or ≤−90%, extremely large (e.large).*

Reference-Bayesian likelihoods of substantial change: *possibly; **likely; ***very likely; and ****most likely.

The symbols *** and **** indicate rejection of the non-superiority or non-inferiority hypothesis (p_*N*__–_ or p_*N*__+_ <0.05 and <0.005, respectively).

*Reference-Bayesian likelihoods of trivial change: ^0^possibly; ^00^likely; ^000^very likely; and ^0000^most likely.*

*The symbols ^000^ and ^0000^ indicate rejection of the superiority and inferiority hypothesis (*p*_–_ and *p*_+_ <0.05 and <0.005, respectively).*

*Effects in bold have adequate precision at the 99% level (*p* < 0.005).*

#### Proportional Error in the Mean Standard Deviation of Power

Proportional error in the SD of power for a 2-SD change in the Reference System was likely trivial for Peach in most stages (2.7% for the 30-s test, and −5.8 to 1.1% for the other stages), small and likely substantial in most stages for EmPower (−43% for the 30-s test, and −24 to −10% for the other stages), small and decisively substantial in most stages for the Concept2 (−53% for the 30-s test, and −29 to −0.4% for the other stages), and moderate and decisively substantial in most stages for Weba (−43% for the 30-s test, and −42 to −23% for the other stages).

#### Between-Unit Differences in the Mean Standard Deviation of Power

Between-unit differences in the mean SD of power are illustrated by the SD bars in Figure 7 (right). Positive between-unit variance for Peach is evident in six stages and was likely or decisively trivial in most of these stages (SDs of 13% for the 30-s test, and 0.6–3.3 for the other stages). Positive between-unit variance for EmPower is evident in three stages and ranged from small to moderate (8.6, 12, and 24% for Stages 1, 5, and 7, respectively) but were unclear. The other stages for Peach and EmPower showed negative variance. Positive between-unit variance was observed in all stages for Weba and was moderate in most stages (25% for the 30-s test, and 19–40% for the other stages); although the estimates all were unclear, the random-effect solutions for Weba units showed that specific units tended to have consistent error across the stages (data not shown). As with the analysis of means, no between-unit differences were possible for the Concept2.

## Discussion

This is the first study to assess the concurrent validity of power output from on-water rowing instrumentation systems. Additionally, the comparison of power from on-water instrumentation systems to that from a Concept2 Model D rowing ergometer had not been investigated previously and provides valuable insight into differences between on- and off-water measures of power in rowing. The devices were assessed over a wide range of intensities and stroke rates, and in an on-water rowing-specific range of motion for Peach, EmPower, and Weba, promoting the applicability of findings from this study for use of these devices on the water. Negative systematic error was evident for all devices in comparison to the Reference System, whereby mean power was lower in all devices than the Reference System; systematic error was of similar magnitude for Peach, Weba, and the Concept2, but was greater in EmPower. Less measurement sensitivity of stroke-to-stroke variations in power were observed in comparison to the Reference System for the Concept2 and Peach, but were negligible in Peach where concurrent variations in power with the Reference System were observed (Figure 6). EmPower and Weba added random error (noise) to stroke-to-stroke variations in power in comparison to the Reference System (Figure 6). There was some evidence of substantial between-unit differences in mean power for Peach, EmPower and Weba, but the SDs representing between-unit differences all were unclear. Between-unit differences were not apparent in the SD of power (i.e., units did not differ in their measurement sensitivity or noise) for Peach and EmPower, whereas Weba units differed in their amount of noise, but again all the SDs were unclear.

### Systematic Error

Differences from the Reference System in device mean power inform potential systematic error for the given device. It should be noted that the Reference System does not provide a criterion measure of power, rather, concurrent validity in comparison to the Reference System is reported. Calculation of power from the Reference System includes a correction for force relative to oar angle (see section “Materials and Methods”), where error for the Reference System may have been introduced. The consistent ∼10% difference in mean power for Peach, Weba, and the Concept2 from the Reference System may therefore reflect positive systematic error for the Reference System whereby true power is closer to that of Peach, Weba, and the Concept2. Only the Concept2 has been investigated previously for its validity of power output, where negative systematic error of ∼7% was found in comparison to instrumentation similar to that used in the current study (Boyas et al., 2006). Most of the apparent systematic error in the current study may therefore be coming from the devices rather than the Reference System. The greater negative systematic error for EmPower in comparison to the other devices may be related to the stepped pattern in power output sometimes evident during testing (as illustrated in Figure 6 for EmPower).

Differences from the Concept2 in mean power for Peach, EmPower, and Weba can be used by practitioners to inform expected differences between on- and off-water power outputs, and the extent to which differences are related to device measurement or the technical demand of on-water rowing. Power output at high intensities is lower on-water than on a Concept2 ergometer over the same test duration (Vogler et al., 2010). Over a 2000-m time trial, a ∼15% lower mean power has been observed on-water with Peach than for the same test on a Concept2 ergometer (personal observations of two of the authors). However, differences in mean power between Peach and the Concept2 in the current study were only ∼1% for high intensities (Stages 6 and 7). The smaller difference between the Peach and Concept2 at high intensities in the current study than that observed when these devices are used in the field suggests the systematic error differences between the two devices contribute only a small portion of the overall difference between on- and off-water power. The remaining discrepancy between on- and off-water power that is not related to systematic error differences between the devices may therefore reflect a reduction in the power that is applied on-water in comparison to that applied on a Concept2 ergometer. The differing technical demand of on-water rowing in comparison to rowing on an ergometer (Kleshnev, 2005) may constrain the power applied during high intensity efforts on-water, as demands such as the entry and exit of the oar from the water will influence the power applied on water, but do not contribute to a rowing stroke on a Concept2 ergometer.

Readers may notice that Concept2 power reported for the maximal stages (30-s test and Stage 7) is relatively low for trained rowers. The low Concept2 power recorded corresponds with the observed ∼14% lower Concept2 power values on the Swingulator at the same heart rate, and the higher perceived effort reported by athletes for the same given power in comparison to those on the Concept2 when used independently from the Swingulator (authors own observations, data not shown). The added resistance through the Swingulator’s pulley system and the indirect force application at the oar (as opposed to in line with the chain when applied at the Concept2 handle) may explain the lower maximal Concept2 power achieved when on the Swingulator.

#### Proportional Error in Systematic Error

The systematic error magnitudes reported are relative to the corresponding mean power per stage, and therefore may differ at different magnitudes of power. Proportional error (the change in systematic error for a 10% change in mean power) represents the relationship between power and systematic error, enabling practitioners to estimate the relative systematic error for a given power output, such as during a race start or short high-intensity intervals where power output is very high. Proportional error was evident only for Peach and EmPower, and only for the 30-s test, where it was negative. Negative proportional error would produce a greater underestimation of power in comparison to the Reference System at higher power outputs, which is evident in the greater negative systematic error observed for the 30-s test in comparison to the other stages for Peach and EmPower in Table 1. It is possible that the proportional error for the 30-s test in Peach and EmPower is related to the location of measurement of these devices at the oarlock (Figure 1), as proportional error was consistently trivial for Weba and the Concept2 (which were located at different positions on the Swingulator system). Although rowing performance tests rarely encompass durations as short as 30 s, practitioners should be aware of the negative proportional error introduced by Peach and EmPower at very high power outputs.

#### Between-Unit Differences in Systematic Error

Based on the between-unit differences in mean power, use of the same unit for repeated measurements or comparing rowers is recommended to remove any potential error introduced by individual units. Although between-unit differences were unclear, specific units showed consistent error across the stages, indicating real differences exist between units. Furthermore, the magnitude of between-unit differences ranged up to large or extremely large for all devices. The unclear effects representing between-unit differences is due not only to the limited number of units assessed for each device, but also to the substantial session-to-session TEM (the random variation in mean power arising between sessions), which reduced the ability to partition error to specific units.

#### Between-Session Error and Between-Participant Error

Differences in mean power between sessions, representing the overall TEM, was partitioned *via* random effects into error introduced by the Reference System across all devices in each session (∼1%), error arising from different participants with each device (Peach ∼3.5%, EmPower ∼−8%, Weba ∼3%, and Concept2 ∼2%), and the residual TEM (i.e., the error introduced by each device in each session; Peach ∼4.5%, EmPower ∼19%, Weba ∼9%, and Concept2 ∼1.5%). Together the error introduced by these three sources (the Reference System, that for different participants, and the residual TEM) reflect the total error introduced between sessions. Although mostly unclear, the error introduced by the Reference System between sessions was smallest of these three random effects and would represent only a small fraction of the overall TEM. The Reference System was therefore reliable relative to the other devices. Although the Reference System is not a criterion or gold-standard measure of power in the current study (rather it provides a comparative measure for assessing the concurrent validity of the other devices), the error arising from a criterion measure is an important component of the total error observed in validity studies that is often overlooked, and should be considered in future research examining device validity.

The extent to which device error differed between participants was generally unclear, but at least this sample may provide insight into the effect of different rowing styles on device measurement. The positive between-participant differences for Peach, Weba, and the Concept2 may reflect differences in the error introduced by these devices when measuring power from differing rowing styles. Differences between participants in their pattern of force application, catch, and finish angles (or chain position in the case of the Concept2), drive phase durations, or recovery phase durations could be factors contributing to between-participant differences in systematic error. However, further research is needed to better understand the differences in device error arising between participants and whether rowing style and device error are related. The negative between-participant differences for EmPower imply that more error is introduced when testing the same participant (i.e., the noise added to stroke-to-stroke variations in power) than when testing different participants, which is likely related to the stepped pattern in power output occasionally occurring within a stage for EmPower (as illustrated in Figure 6).

The correlations between the residuals supported the interpretation of the residuals as TEM arising independently in each device between sessions. In reliability studies, TEM combines with biological variability (e.g., variability in the power a participant can produce between testing sessions) to give the typical or standard error of measurement in such studies. The residual technical error observed here should therefore be smaller than the typical error observed elsewhere in reliability studies. However, the ∼4.5% TEM observed for Peach is larger than the typical (standard) error of measurement of 1.3–2.2% found for Peach between three 500-m trials in elite scullers (Coker, 2010). The ∼1.5% TEM observed for the Concept2 lies within the range of the 1.3 and 2.8% standard error of measurement values reported for 2000- and 500-m test distances on the Concept2 (Soper and Hume, 2004), but would allow for little biological variability between tests. It is possible that the stationary testing set-up on the Swingulator in the present study could contribute to technical error in some way, at least for Peach and the Concept2, that would not arise when the devices are used as intended, either on-water (in the case of Peach, and possibly also EmPower and Weba), or without attachment to the Swingulator (in the case of the Concept2).

### Mean Standard Deviation of Power, Representing Measurement Sensitivity to Stroke-to-Stroke Variations in Power (Random Error)

The shallow “∪” shape across Stages 1–7 illustrated by the Reference System in its mean SD of power in Figure 7 (right) demonstrates that the participants’ true stroke-to-stroke variability in power output decreased from Stages 1–6. The reduction in variability, particularly over Stages 1–3, is likely due to the difficulty associated with maintaining a consistent power output when the prescribed power is easier than the participants are familiar with. The increase in participant variability in Stage 7 may reflect pacing strategy in this maximal stage, such as a fast start and fast finish, or the inability to maintain a desired target power output. The least stroke-to-stroke variability was evident in the 30-s test (Stage 0), which was likely due to the short test duration, where pacing strategy and fatigue have limited contribution.

The positive differences from the Reference System in the mean SD of power for EmPower and Weba represent random error (noise) in the signal output of these devices. The small to moderate random errors for EmPower would produce modest attenuation of rowing performance predicted by power per stroke for submaximal and maximal intensities over 4 min, but a considerable attenuation of effects for maximal intensities over short durations (∼30 s). The Weba would produce considerable attenuation of rowing performance predicted by power per stroke over all intensities assessed in this study. These attenuations would reduce the ability to detect true effects with power per stroke as a predictor.

Peach appeared to closely follow the stroke-stroke variation in power measured by the Reference System (as shown in Figure 6), although the analysis of the mean SD of power indicated a small amount of measurement sensitivity was lost in comparison to the Reference System. The mostly trivial difference in measurement sensitivity would result in little attenuation of relationships between power per stroke and rowing performance. Future research investigating individual stroke power (if the individual strokes could be aligned consistently) would enable the partitioning of stroke-to-stroke variation in power into the variation arising from the participant, random error (if any) in the Reference System, and any lack of measurement sensitivity (as negative variance) in Peach.

The Concept2 demonstrated a reduced measurement sensitivity in comparison to the Reference System, which improved from Stages 1 to 7, whereas considerable random error was apparent for the 30-s test. Inspection of the stroke-to-stroke data for the Concept2 revealed greater differences from the Reference System over the first ∼5 strokes due to a gradual increase in power from the Concept2 at the start of each stage. These findings are consistent with those of Boyas et al. (2006), who found a reduction in the magnitude of negative systematic error for the Concept2 when they excluded the first three strokes from analysis. The gradual increase in power demonstrated by the Concept2 at the start of each stage (when the flywheel is stationary) likely reflects the increase in flywheel velocity due to the inertia of the flywheel, given that the acceleration of the flywheel is used to calculate power (Boyas et al., 2006; Dudhia, 2017). The effect of a lack of measurement sensitivity would therefore be reduced in later stages, as initial differences from the Reference System at the start of the stage contribute a smaller proportion of the total number of strokes per stage as stroke rate increases. The lack of measurement sensitivity observed for the Concept2 would result in small attenuations of rowing performance predicted by power per stroke.

#### Proportional Error in the Mean Standard Deviation of Power

The mostly trivial proportional error in the SD of power for Peach showed that there was reasonable consistency in the variation of power per stroke in comparison to the Reference System (as illustrated in Figure 6). The negative proportional error in the SD of power for EmPower and Weba represents a reduction in the magnitude of noise introduced to stroke-to-stroke measures of power by these devices when true (Reference System) stroke-to-stroke variation is higher. The negative proportional error observed for the Concept2 probably represents a decrease in measurement sensitivity at higher values of stroke-to-stroke variation, which will have some explanation in terms of the detection of fluctuations in flywheel velocity.

#### Between-Unit Differences in the Mean Standard Deviation of Power

The occurrence of both positive and negative variance for between-unit differences in the mean SDs for Peach and EmPower likely arise from sampling variation, whereby a true variance of practically zero can be expected to produce some positive and some negative estimates of variance. The positive and negative between-unit variances in the mean SDs observed across the stages for Peach and EmPower are therefore consistent with no real differences between the units in their measurement of stroke-to-stroke SD. Conversely, the consistency observed in the magnitude of positive variance across the stages is evidence of real differences between Weba units, notwithstanding the uncertainty of the effects. Use of the same Weba unit for repeated measurements of power per stroke would remove any potential error introduced by between-unit differences, although the magnitude of random error added by Weba to stroke-to-stroke measurements of power (as illustrated in Figure 7, right) is such that Weba is not recommended for the assessment of power per stroke. Some Weba units might also introduce substantial random error into the measurement of mean power in a 2000-m time trial; for example, if the SD of power per stroke for a unit was 16%, the error in the mean of ∼256 strokes in the trial would be 16/√256 = 1%, which represents substantial error.

### Practical Applications

#### Peach

• Practitioners should be aware that power output measured by Peach is likely lower than that performed by the rower by ∼10%, but up to ∼17% at maximal power outputs over short (30 s) durations.
• Power measured by Peach is close to that of the Concept2 (within 2%), but differences of up to 6% exist between the two devices at power outputs below ∼150 W. Differences greater than 2% in power between Peach and Concept2 observed by practitioners therefore likely reflect differences in the application of power relating to the increased technical demand in on-water rowing.
• The TEM for Peach was ∼5% which represents large to very large errors being introduced between sessions. Negligible session-to-session reliability is represented by TEM values of <0.5%.
• Peach can be used with confidence for assessments of stroke-to-stroke power and of relationships between power and rowing performance, given its negligible lack of measurement sensitivity (∼−6% difference from the Reference System in the mean SD of power, and up to 16% at maximal efforts over 30 s).

#### EmPower

• Practitioners should be aware that power measured with EmPower devices may be substantially lower (∼25%) than when measured with Peach, Weba, or Concept2 devices. It is therefore advisable that practitioners use the same device when comparing measures of power output, particularly when using EmPower.
• The TEM for EmPower was ∼18% which represents extremely large errors being introduced between sessions. Negligible session-to-session reliability is represented by TEM values of <0.5%.
• EmPower is best used to assess mean power rather than power per stroke owing to the noise in its signal output, which was represented by random error estimates of ∼15% and up to 57% at maximal efforts over 30 s. Negligible random error magnitudes are <11% for stroke-to-stroke measures of power.

#### Weba

• Practitioners should be aware that power output measured by Weba is likely lower than that performed by the rower by ∼10%, but is similar (within ∼5%) to that of Peach and the Concept2.
• The TEM for Weba was ∼10% which represents extremely large errors being introduced between sessions. Negligible session-to-session reliability is represented by TEM values of <0.5%.
• Weba is best used to assess mean power rather than power per stroke owing to the noise in its signal output, which was represented by random error estimates of 61–139%. Negligible random error magnitudes are <11% for stroke-to-stroke measures of power.

#### Concept2

• Practitioners should be aware that power output measured by Concept2 is likely lower than that performed by the rower by ∼10%, but is similar (within ∼5%) to that of Peach and Weba.
• The TEM for Concept2 was ∼1.5% and was lower than that for Peach, Weba, and EmPower. Negligible session-to-session reliability is represented by TEM values of <0.5%, nonetheless the magnitude of error introduced by the Concept2 between sessions is only small.
• Concept2 measurement sensitivity for the assessment of stroke-to-stroke power is ∼20% lower in comparison to the Reference System. When assessing stroke-to-stroke power practitioners should exclude the first ∼5 strokes or use tests involving rolling starts to account for the greater negative offset in power associated with stationary starts on the Concept2.

## Conclusion

Mean power was found to be lower in comparison to the Reference System for all devices. Magnitudes of negative systematic error were similar for Peach, Weba, and the Concept2, but larger for EmPower. Stroke-to-stroke variations in power were consistent between Peach and the Reference System, but a small reduction in measurement sensitivity was evident for the Concept2, whereas EmPower and Weba introduced noise. There was some evidence of between-unit differences in mean power for Peach, EmPower, and Weba, and in the SD of power (stroke-to-stroke fluctuations) for Weba. The findings of this study can be used by practitioners to inform the interpretation of meaningful change in measures of power when using the devices assessed.

## Data Availability Statement

The datasets presented in this study can be found in online repositories. The names of the repository/repositories and accession number(s) can be found below: Open Science Framework at doi: 10.17605/OSF.IO/HQ4W2, https://osf.io/hq4w2/.

## Ethics Statement

The studies involving human participants were reviewed and approved by the Victoria University Human Research Ethics Committee (VUHREC). The patients/participants provided their written informed consent to participate in this study.

## Author Contributions

AH, RA, RS, WH, and KB designed the study and wrote the manuscript. AH collected the study data. VR contributed to data analyses. WH performed the statistical analyses. All authors contributed to the article and approved the submitted version.

## Conflict of Interest

The authors declare that the research was conducted in the absence of any commercial or financial relationships that could be construed as a potential conflict of interest.

## Publisher’s Note

All claims expressed in this article are solely those of the authors and do not necessarily represent those of their affiliated organizations, or those of the publisher, the editors and the reviewers. Any product that may be evaluated in this article, or claim that may be made by its manufacturer, is not guaranteed or endorsed by the publisher.
